# Preparation and characterization of acetylsalicylic acid/chitosan nanoparticles and its antithrombotic effects

**DOI:** 10.1080/15685551.2018.1534317

**Published:** 2018-10-16

**Authors:** Shang Luo, Hua Man, Xile Jia, Yuanyuan Li, Aihong Pan, Xuecheng Zhang, Yimin Song

**Affiliations:** a Colloge of Chemical Engineering, Qingdao University of Science and Technology, Qingdao, P.R. China; b Colloge of Marines Life Science, Ocean University of China, Qingdao, P.R. China

**Keywords:** ASA, CSnanoparticle, interpolymer complexation method, antithrombotic effect

## Abstract

Chitosan (CS)-acetylsalicylic acid (ASA) nanoparticles, which are well dispersed and stable in aqueous solution, have been prepared by interpolymer complexation of ASA in CS solution. The physicochemical properties of nanoparticles were investigated by using FT-IR, ^1^H NMR, scanning electron microscope（SEM）, dynamic light scattering, and UV spectrophotometer. It was found that the carboxyl group of the ASA had firmly integrated on the amino group of CS and the ASA-CS nanoparticles were almost spherical in shape with an average diameter of less than (79.3 ± 24.6) nm in high reproducibility and showed high chemical stability against environmental changes. It was also found that the prepared nanoparticles carried a positive charge and showed the size in the range from 700 to 150 nm. The surface structure and zeta potential of nanoparticles can be controlled by different preparation processes. The factor experiment results indicated that the ASA-CS nanoparticles had satisfactory loading capacity (LC) and encapsulation efficiency (EE), 27.27% and 46.88% (data not shown), respectively. The experiments of in *vitro* ASA release showed that these nanoparticles provided a sustained and pH-dependent drug release manner, and the release behavior was influenced by the pH value of the medium. Preliminary pharmacology experiment exhibited prolonged circulation and higher bioavailability than that of ASA. All the results indicated that ASA/CS nanoparticles may have promising pharmaceutical application, and further pharmacological research is needed to confirm these beneficial effects.

## 1. Introduction

Acute myocardial infarction (AMI) and thromboembolic disease are a major cause of morbidity and mortality worldwide, so the development of effective drugs for the prevention and treatment of such diseases has increasingly attracted worldwide attention. Acetylsalicylic acid (ASA) had significant anti-platelet aggregation function [1] and it had been used for the prevention and treatment of AMI and thrombosis diseases from 1970s [2]. Evidence-based studies had proved that ASA could reduce the incidence rate of 25% of heart and brain and peripheral thromboembolic disease [3,4]. Especially for myocardial infarction and stroke, long-term use of the low-dose acetylsalicylic acid (ASA) could significantly reduce the incidence of this diseases. However, the adverse effects of oral ASA tablets such as gastrointestinal disorders and bleeding often led to treatment discontinuation. Furthermore, many studies found that some patients would get aspirin resistance (AR) or aspirin failure (AF) [5]. All of these limited the application of ASA. To improve the pharmacological properties of ASA, lots of research work has been done around the world [6–9], but little progress has been made. Therefore, how to improve the clinical efficacy, reduce adverse reactions, expand the scope of application and overcome the AR phenomenon of ASA is still one of the hottest points in field of medicine.

Chitosan (CS) is a species of highly promising drug carrier. Due to its polymeric cationic characteristics, CS can interact with negatively charged molecules or polymers, and thus CS nanoparticles can be formulated by ionic interaction between the cationic CS and anionic counter ions leading to interpolymer linkages [10]. It was found that the preparation of nanoparticles could be simple, non-toxic, without organic solvents, and could be carried out at room temperature, and which had been applied in preparing micro/nanoparticles for the encapsulation of drugs or biological substances as carrier and stabilizer [11–15]. Especially in the last few years, it has been recognized that chemical modification of lead compounds with active groups in CS and its derivatives could improve the solubility, efficacy, and reduce the side effects. It was also found that CS had a synergistic effect with the precursor drug, including the derivatives of monosaccharide, disaccharide, oligosaccharide, and polysaccharide [16]. On these ground, the chemical modification of ASA by CS might be one of the important measures to develop an ideal drug for the prevention and treatment of cardiovascular diseases.

The preparation and application of chitosan/acetylsalicylic acid complex nanoparticles has not been reported yet. In the present study, acetylsalicylic acid/chitosan (ASA-CS) nanoparticles were prepared by interpolymer complexation method. The physicochemical properties of nanoparticles were investigated and the structure of the ASA-CS nanoparticles were characterized. The influence of different preparation condition on the ASA-CS nanoparticles loading capacity (LC) and encapsulation efficiency (EE) were explored. Moreover, the release behavior of ASA-CS nanoparticles in medium with different pH values, chemical stability against environmental changes and the antithrombotic effect in vitro were studied.

## 2. Materials and methods

### 2.1 Materials

Chitosan (CS) are prepared with the hydrogen peroxide hydrolysis method by ourselves and the molecular weight of CS are 1200, 880, 530, 320, 38, 22 kDa respectively [17]. The deacetylation degree (DD) of CS were measured by acid-base titration. The results showed that there were no significant difference among different molecular weight of CS，and DD of CS was 85%. ASA-CS conjugates were prepared by ourselves with different molecular weight (*M*_w_) of CS ranging from 22 ~ 1200 kDa. Acetylsalicylic acid was purchased from Sinopharm Chemical Reagent Co., Ltd, China. ^125^I-thromboxane B_2_ and ^125^I-6-keto-PG F_1a_ RIA kits were commercially obtained from Suzhou Medical College (Suzhou, People’s Republic of China). Thrombin, adenosine diphosphate (ADP) and bovine serum albumin (BSA) and apyrase were all purchased from Sigma Co. (St. Louis, MO). All other solvents and chemicals were without further purification. Distilled or deionized water was used in all experiments.

### 2.2 Preparation of ASA-CS nanoparticles

**Figure 1.**
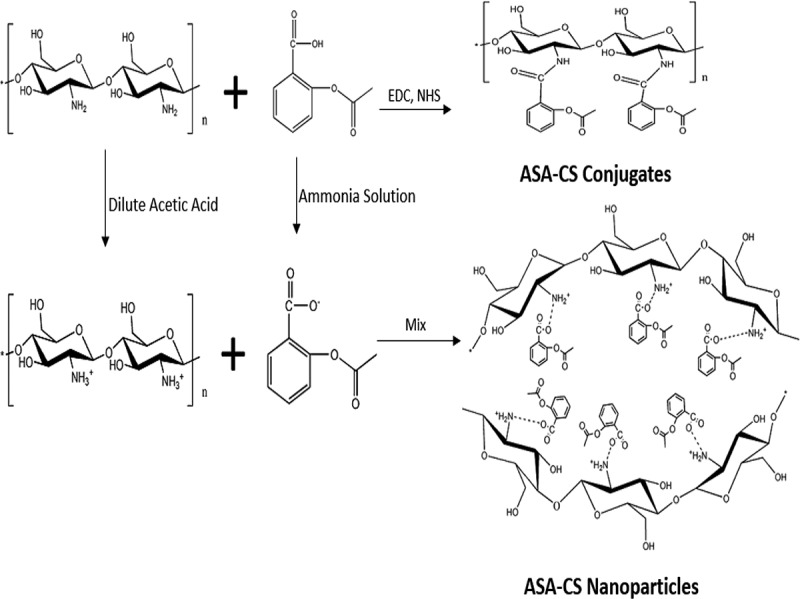
The reaction schemes of CS and ASA.

The reaction schemes of ASA and CS were shown in Figure 1. ASA-CS nanoparticles were prepared based on the interpolymer complexation method. The electrostatic interaction between the amino group of CS and carboxyl group of ASA was the driving force [10]. CS solution was firstly prepared, and then ASA was further conjugated onto CS *via* carbodiimide chemistry. CS solution (3%, w/v) was prepared by dissolving 3 g CS in 100 mL dilute acetic acid (3%, v/v) at room temperature under magnetic stirring. ASA ammonium salt solution (3%, w/v) was prepared in the same way instead dilute acetic acid (3%, v/v) of 10%-25% ammonia solution. At different reaction temperatures, CS solutions of different molecular weights (with different pH values ranging from 3.0 to 5.5) were mixed with ASA ammonium salt solution (ASA/CS molar ratio of 0.25, 0.5, 0.75, 1.0 and 1.5). Then ultracentrifugation (10,010 × g, 0.5 h) was utilized to separate the free ASA. The supernatant was extracted and the precipitation was washed. The operation was repeated three times, collecting supernatants and precipitations. The resulting conjugate products were frozen in a refrigerator (-70 ^o^C) and then freeze-dried using a ALPHA2-4/LSC-16 lyophilizer (MARTIN CHRIST, Germany) with a condenser temperature of -45 ^o^C and inside pressure less than 25 Pa. By addition, the samples used in the following contents were prepared in the optimal conditions unless we said otherwise.

### 2.3 Characterization of ASA-CS nanoparticles

The mean size, size distribution and zeta potential measurements were performed on a Malvern Zetasizer Ver. 6.32 (Malvern Instruments, UK) in deionized water. The surface morphology of the ASA-CS nanoparticles was observed by scanning electron microscope (SEM) (Hitachi, JSM-6700F, Japan). For SEM, the nanoparticles suspensions were spread on a glass plate and dried at room temperature. The dried nanoparticles were coated with gold metal under vacuum and then examined. The chemical structure and complexes formation of CS and drug-loaded CS nanoparticles were analyzed by fourier transform infrared spectroscopy (FT-IR) (Bruker, TENSOR, Germany) and nuclear magnetic resonance (^1^H NMR) (Bruker, NMR500, Germany). The samples for infrared analysis were prepared by grinding the dry specimens with KBr and pressing the mixed powder to form disks.

### 2.4 Stability study of ASA-CS nanoparticles

The same batch of freeze-dried nanoparticle productions was sub-packed in 2 ml ampoule and were subjected for long-term stability studies and accelerated stability studies. Long-term stability studies were carried at 5 ± 2^o^C/60% ± 5% RH, 30 ± 2 ^o^C/65% ± 5% RH. The samples were stored at the above said condition for minimum 10 months and their morphological features and diameter, LC and in vitro release were determined for every 3 months. In the same way, an accelerated stability study was carried out by storing the selected preparations at 40 ± 2^o^C/75% ± 5% RH for about 4 months. The morphological features and diameter, LC, and in vitro drug release behavior of ASA-CS nanoparticle were determined for every 1 months.

### 2.5 In vitro release of drug

The release behavior of ASA from ASA-CS nanoparticles was studied in *vitro* by a dialysis method in different release medium (pH 1.2, 5.5, 6.5, 6.8 and 7.4). The freeze-dried ASA-loaded nanoparticles were placed in dialysis bag (Mwco 6–8 kDa) with solvent of different pH, and incubated at 37 ± 0.1^o^C under stirring. A certain volume of release medium is removed at appropriate intervals and immediately replaced with an equal volume of fresh release solution. The amount of ASA released from the nanoparticles was evaluated by UV spectrophotometer. The ten samples were analyzed at each time point and the calibration curve was made using non-loaded ASA nanoparticles as correction.

### 2.6 Determination of carotid artery thrombosis in rats

Electrical stimulation was delivered to the carotid artery to resulting in a consistent formation of arterial thrombosis [18]. This experiments of carotid artery thrombosis were based on the documented method [19], which was improved by our lab as follows. Under sodium pentobarbital anesthesia (30 mg/kg, *intraperitoneally*), one side carotid artery in rats was separated and hooked with a stimulation electrode and thermo-probe of BT 87–2 experimental thrombosis detector [20] (made in Cardiovascular Laboratory of Inner Mongolia University of Science and Technology). Healthy Wister rats weighing for 256 ± 19 g, female or male not limited, were randomly assigned to 6 groups: control group, ASA group, ASA-Conjugates group, ASA-CS nanoparticles low dosage group, ASA-CS nanoparticles middle dosage group, and ASA-CS nanoparticles high dosage group. Then Stomach of rats was perfused with normal saline (group A), ASA 0.5g/kg (group B), ASA-Conjugates 0.5g/kg (group C), ASA-CS nanoparticles 0.25g/kg (group D), ASA-CS nanoparticles 0.5g/kg (group E), ASA-CS nanoparticles 1g/kg (group F). 15 min latter, they were subjected to sustained electrical stimulation [21] at 1.5 mA intensity for 7 min over the carotid artery area of rats to damage endothelial cells, which resulted in gradually forming mixed thrombus in blood vessel. Then the carotid artery blood flow was blocked by the thrombus, which brought about sudden reduction of carotid artery temperature and the alarm of the instrument. The interval from the start of stimulation on blood vessel to sudden drop of the temperature of carotid artery was defined as the Occlusion Time (OT) recorded by BT 87–2 Experimental Thrombosis Detector. Groups B-F were administrated to drugs by gavage as group (A) and 10 rats were used in experiments at each time in group A-F.

## 3. Results and discussion

### 3.1 FT-IR detection

**Figure 2.**
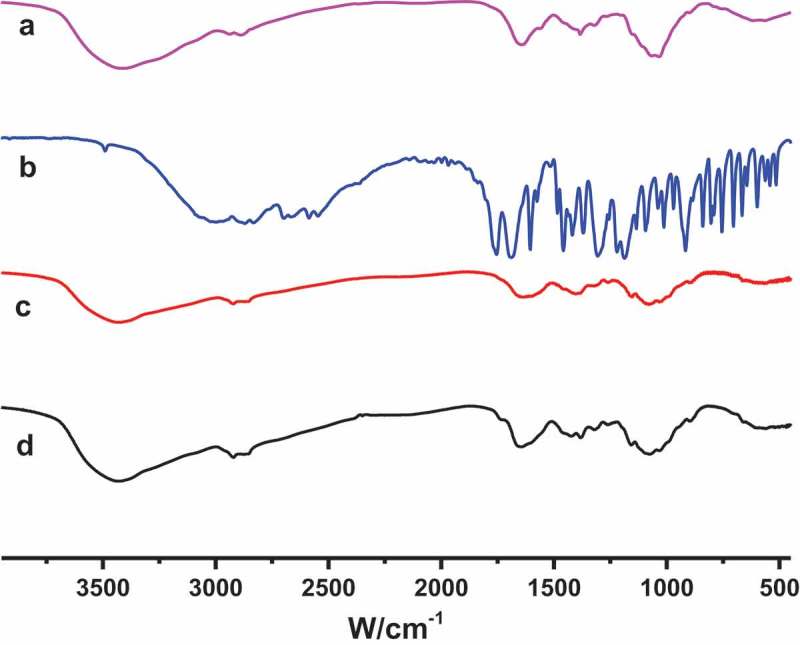
FT-IR of (a) CS, (b) ASA, (c) ASA-CS conjugates and (d) ASA-CS nanoparticles.

The infrared spectrum diagrams of ASA-CS nanoparticles were shown in Figure 2. The results demonstrated the IR spectrum of typical chemical groups of ASA-CS nanoparticles at 3425 cm^−1^ (–OH stretching), 2929 cm^−1^and 2890 cm^−1^ (–CH stretching), 1420 cm^−1^ (–NH_2_ stretching) and 1076cm^−1^ (C–O–C stretching). The absorption of 1688cm^−1^ (carboxyl group absorption peak) moves to 1630cm^−1^. The – NH_2_ bending vibration shifted from 1646 cm^−1^ to 1630 cm^−1^ and a new peak at 1595 cm^−1^ appeared, which indicated that some interaction between ASA and CS have occurred within the nanoparticles. This showed that the ionic bond was formed between acetylsalicylic acid carboxyl and chitosan amino.

### 3.2 NMR spectrometry

**Figure 3.**
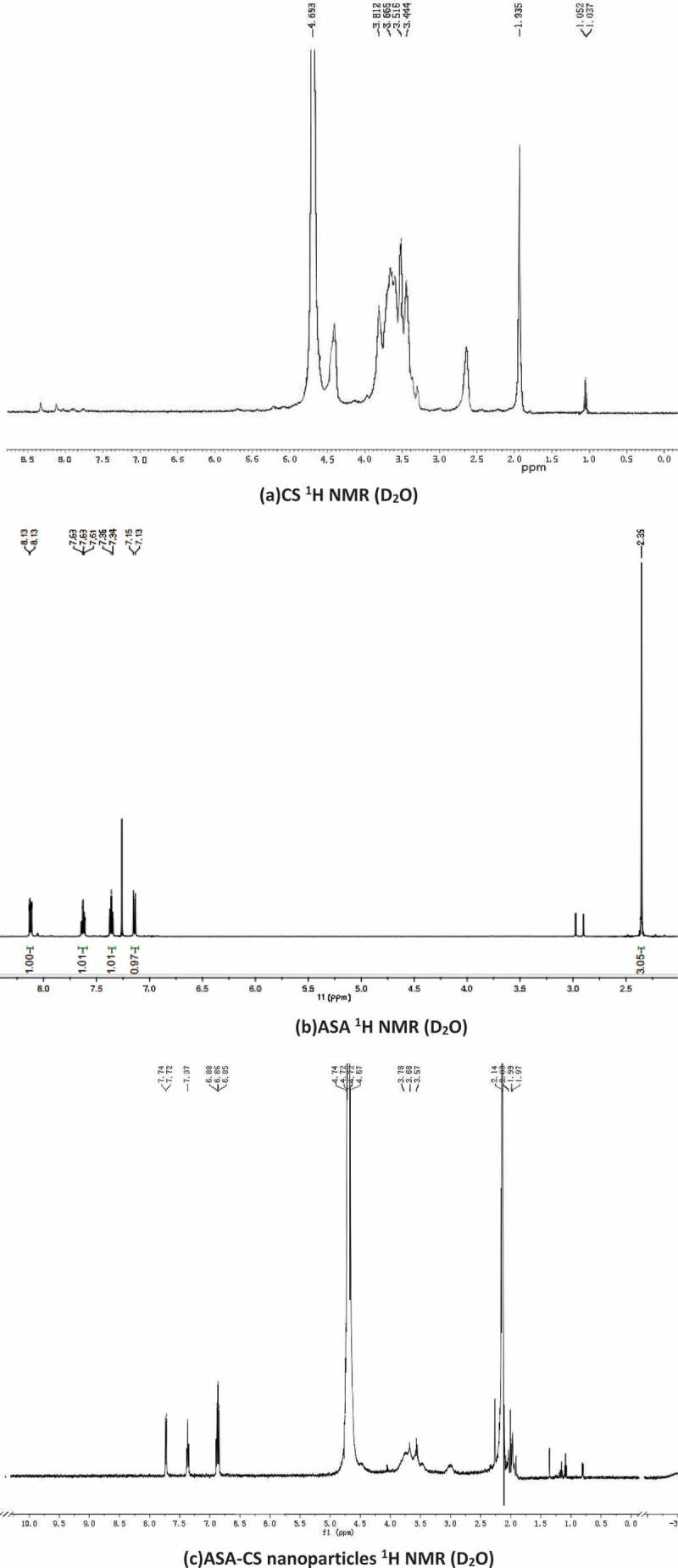
^1^H NMR spectra of (a) CS, (b) ASA, (c) ASA-CS nanoparticles.

The Figure 3 shown the ^1^H NMR spectra of three materials (b) and (c) were similar on the whole but not for (a). Peak at 2.0 was assigned to the methyl hydrogen of N-acetylglucosamine. The ring protons (H-3,4,5,6,6ʹ) of CS were considered to resonate at 3.0–4.0. In the spectrum of ASA-CS nanoparticles, the new proton peaks at 0.90 and 1.1 were assigned to CH_3_ and CH_2_ of the ASA residue, confirming the coupling of ASA to CS. There were 3 aromatic hydrogens signals at 6.85–7.75. We conjectured that 7.749 and 7.733 were acyl ortho hydrogen signal peak of acetylsalicylic acid. 7.396, 7.381 and 7.365 were acyl para hydrogen signal peak of acetylsalicylic acid. 6.897 and 6.851 were hydroxyl ortho hydrogen signal peak of acetylsalicylic acid. 6.882 and 6.867 were hydroxyl para hydrogen signal peak of acetylsalicylic acid. Due to adding deuteroxide to deuterated reagent, the active hydrogen signal peak disappeared. Comparison of the ^1^H NMR data among acetylsalicylic acid, chitosan and ASA-CS nanoparticles, it showed that the acetylsalicylic acid acyl had replaced the -NH of chitosan，which indicated that the acetylsalicylic acid acyl had combined into the chitosan amino.

### 3.3 Morphology measurements

**Figure 4.**
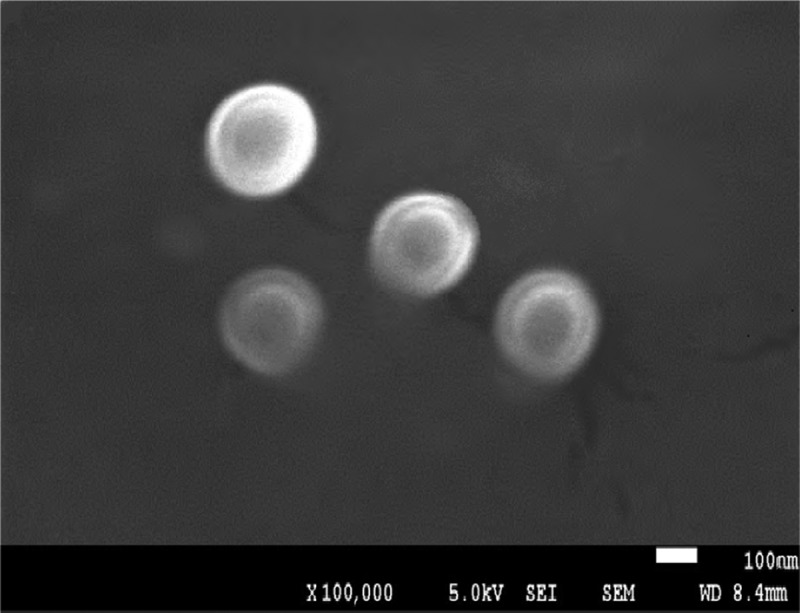
SEM of ASA/CS nanoparticles.

SEM photos of the ASA-CS nanoparticles was shown in Figure 4. Using the properties of amino group on the chitosan, which was cationic in dilute acid，and carboxyl group on the ASA, which was anionic in dilute alkaline solution, polyelectrolyte compounds with intermolecular force were synthesized by CS and ASA in our laboratory. More specific, protonated amines of CS chain would gradually connect to the carboxyl of ASA chain with coulomb force in molecules and between molecules, at the same time shrinking and crimping CS chain, eventually leading to production and nanocrystallization of CS in the process of reaction [22]. The previous FT-IR also proved that this process was not only hydrogen bonding and Van der waals forces, but also the formation of ionic bonds. Based on the analysis above we could conclude as from Figure 4 that nanoparticles were nearly spherical, smooth on the surface, dense and had no crack and hole.

### 3.4 Particle size and zeta potential analysis

**Figure 5.**
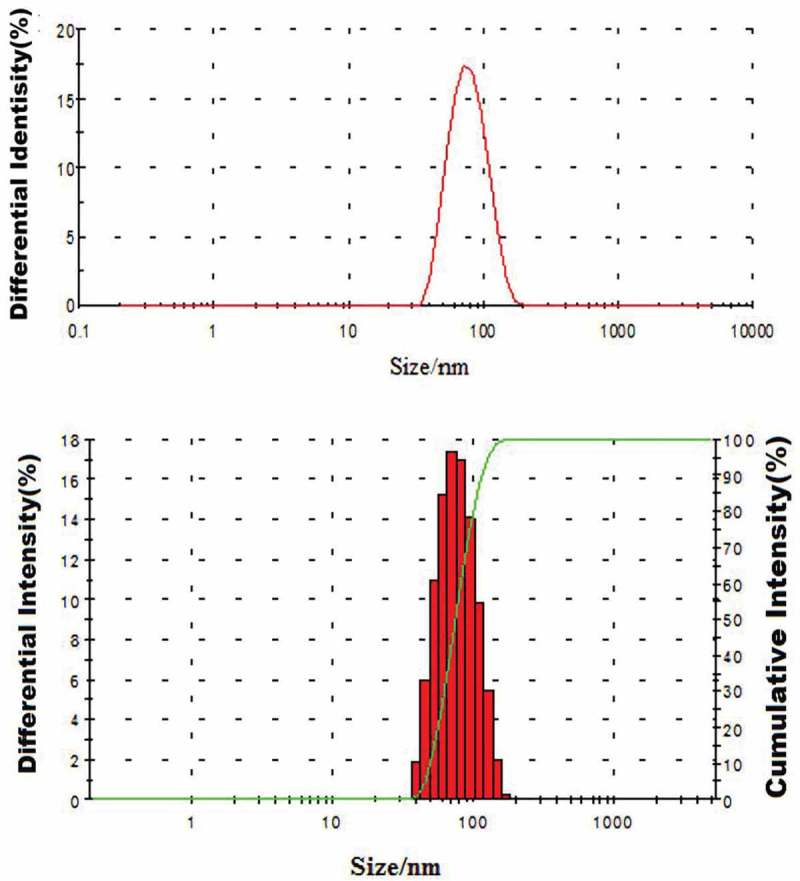
Size distribution of ASA-CS nanoparticles.

The particle size was a crucial determinant of nanoparticles, which meant that particle size should be controlled in a certain range. It was found that the particle size was affected by a series of factors such as *M*_w_ of CS, pH value of medium, and reaction temperature, etc. as shown in Tables 1, 2, 3 and 4. It was also found that within a certain range，the particle size of ASA-CS nanoparticles increased with the increase of *M*_w_ of chitosan. Meanwhile, the increase of the pH value of medium and reaction temperature would cause the decrease of the particle size of ASA-CS nanoparticles. The results above could be explained by the fact that unit molar CS with high *M*_w_ has more monosaccharide units and carries more amino acids, which makes it easy for positively CS with high *M*_w_ to combine more ASA and aggregate into larger nanoparticles. While, pH and reaction temperature were contrary to *M*_w_, which were due to the reason that the higher pH value of medium could reduce the solubility of CS and the higher temperature would destroy the crystalline structure of CS. By addition, molar ratio of ASA and CS had a slight influence on the size of ASA-CS nanoparticles and the influence of these factors on zeta potential were opposite to the size of ASA-CS nanoparticles, which might be related to the decrease or increase of the positive charges of CS. As shown in the Figure 5, the final molecule weight of the material was 32 × 10^4^ Da. The size distribution analysis results of ASA-CS nanoparticles presented the consistency of the results of electron micro-scopy examination. The average size was about 79.3 ± 24.6 nm. Therefore, the conclusion that narrowing particle size distribution of the ASA-CS nanoparticles, which was helpful to enhancing drug loading property of nanoparticles, was reached.

**Table 1.** Effect of CS *Mw* on EE and LC of ASA-CS nanoparticles (mean± SD, n = 3).

n(ASA):n(CS)	*M*_w_ (× 10^4^ Da)	EE(%)	LC (%)	Diameter(nm)	Zeta-potential(mV)	PDI
0.75	120	25.47	17.69	142.17 ± 20.56	42.29 ± 0.34	0.337
0.75	88	39.48	22.41	101.55 ± 18.36	40.96 ± 0.95	0.303
0.75	53	44.76	26.55	86.78 ± 21.60	40.43 ± 0.73	0.216
0.75	32	46.67	27.12	79.52 ± 22.13	39.62 ± 0.86	0.104
0.75	3.8	45.81	23.94	81.66 ± 19.67	36.24 ± 1.02	0.153
0.75	2.2	45.09	24.07	78.71 ± 21.35	31.03 ± 0.63	0.121

Experiment conditions: temperature: 55 – 60 ℃, reaction time: 4 h，pH = 5.

**Table 2.** Effect of pH on EE and LC of ASA-CS nanoparticles (mean± SD, n = 3).

pH	*M*_w_ (× 10^4^Da)	EE (%)	LC (%)	Diameter(nm)	Zeta-potential(mV)	PDI
3.0	32	35.17	17.32	72.37 ± 19.31	40.19 ± 0.66	0.217
3.5	32	40.68	20.07	71.97 ± 20.53	38.11 ± 0.51	0.221
4.0	32	43.55	22.88	72.96 ± 21.77	37.31 ± 0.42	0.241
4.5	32	45.91	25.19	76.41 ± 19.86	35.72 ± 0.71	0.207
5.0	32	46.97	27.88	80.65 ± 22.21	31.16 ± 0.56	0.189
5.5	32	42.31	23.49	125.27 ± 20.10	28.77 ± 0.91	0.303

Experiment conditions: n(ASA)：n(CS) = 0.75，reaction temperature: 55–60℃，reaction time: 4 h

**Table 3.** Effect of reaction temperature on EE and LC of ASA-CS nanoparticles (mean± SD, n = 3).

Reaction temperature	*M*_w_ (× 10^4^Da)	EE (%)	LC(%)	Diameter(nm)	Zeta-potential(mV)	PDI
35–40	32	39.18	23.67	70.37 ± 19.77	41.73 ± 0.91	0.235
45–50	32	41.91	23.91	74.99 ± 22.51	36.67 ± 0.62	0.317
55–60	32	47.11	26.25	79.19 ± 18.66	35.19 ± 0.71	0.274
65–70	32	44.76	27.03	89.37 ± 21.55	32.44 ± 0.67	0.301
75–80	32	42.68	26.81	103.78 ± 21.75	30.81 ± 0.34	0.231

Experiment conditions: n(ASA):n(CS) = 0.75，reaction time:T = 3h

**Table 4.** Effect of reactant molar ratio on EE and LC of ASA-CS nanoparticles (mean± SD, n = 3).

n(ASA):n (CS)	*M*_w_ (× 10^4^Da)	EE(%)	LC (%)	Diameter(nm)	Zeta-potential(mV)	PDI
0.25	32	19.65	11.65	79.12 ± 16.55	40.91 ± 0.92	0.203
0.50	32	40.41	21.97	80.37 ± 19.31	38.64 ± 0.61	0.246
0.75	32	46.76	27.81	78.94 ± 17.61	36.73 ± 0.83	0.164
1.0	32	47.23	28.19	79.68 ± 18.33	31.89 ± 0.99	0.222
1.5	32	47.03	28.10	81.01 ± 19.38	30.01 ± 0.87	0.219

Experiment conditions: reaction temperature: 55–60 ℃, reaction time:T = 3h, pH = 5.

### 3.5 Influence of different experimental conditions on the properties of ASA-CS nanoparticles

#### 3.5.1 Influence of CS m_w_ on EE and LC of ASA-CS nanoparticles

This part adopted the single factor control variable method, including the four aspects: pH, chitosan molecular weight, reaction temperature, the ratio of n(ASA):n (CS), to explore the optimal experimental conditions preparation of ASA–CS nanoparticles. And using the following method determined the encapsulation efficiency (EE) and loading capacity (LC).

The amount of free ASA was determined by a UV-Visible spectrophotometer (Shimadzu, UV-2450, Japan) at λ max after suitable dilution. A calibration curve was pre-established under the same conditions. The EE and LC were calculated by the following equations:

EE = *W_2_/W_1_ *× 100%

LC = *W_2_/W *× 100%

In the above equations, *W* is the weight of nanoparticle powders. *W_1_* is the total amount of ASA. *W_2_* is the encapsulated ASA.

The influences of different chitosan *M*_w_ on chitosan nanoparticles EE and LC were shown in Table 1. We could conclude that compared with the LC of ASA-CS nanoparticles that was prepared by chitosan which *M*_w_ was 120 × 10^4^ Da，it was lower than that was prepared by chitosan which *M*_w_ was 32 × 10^4^ Da on the condition of the same feed ratio, being inversely proportional to its *M*_w_. However, the EE and LC of ASA-CS nanoparticles that was prepared by chitosan which *M*_w_ respectively was 2.2 × 10^4^ Da, 3.8 × 10^4^ Da, 32 × 10^4^ Da didn’t have obvious differences. It showed that within a certain range of the CS *M*_w_, adopting this method to prepare ASA-CS nanoparticles, different *M*_w_ of chitosan had no obvious influence on EE and the LC of ASA-CS nanoparticles.

#### 3.5.2 Influence of ph value in the medium on EE and LC of ASA-CS nanoparticles

The influences of pH value in the medium on the EE and LC of ASA-CS nanoparticles were shown in Table 2. It could be concluded from the Table 2 that on the condition that the pH value was less than 4 the EE and drug-loadings rate of ASA-CS nanoparticles increased with the pH value increasing. The nanoparticles had a higher EE at pH 4.0, the reason for this could be that the formulation pH value was near the pKa of ASA (pKa = 3.5) and the protonation of CS was enhanced at low pH value. But when the pH value was more than 4, the EE and LC of ASA-CS nanoparticles increased very slowly and almost did not change. At pH 5.0, EE and LC of ASA-CS nanoparticles got maximum value. However at pH 5.5, EE and LC of ASA-CS nanoparticles were reduced instead. So it was advisable to choose pH 5.

#### 3.5.3 Influence of reaction temperature on EE and LC of ASA-CS nanoparticles

The influences of reaction temperature on the EE and LC of ASA-CS nanoparticles were shown in Table 3. Based on the results, we could conclude that the EE and LC of ASA-CS nanoparticles increased distinctly with the increase of reaction temperature, which ranged from 35–60℃. But at 65-80^o^C, the EE and LC of ASA-CS nanoparticles declined evidently with the increase of reaction temperature. So it is suggested that the preparation of ASA-CS nanoparticles should strictly control the reaction temperature between 55 ~ 60℃. In the meantime, as could be seen from the experimental data, the influence of pH value of medium on the EE and LC was relatively smaller than that of reaction temperature.

#### 3.5.4 Influence of molar ratio between ASA and CS on EE and LC of ASA-CS nanoparticles

The influences of molar ratio between ASA and CS on the EE and LC of the ASA-CS nanoparticles were shown in Table 4. We could conclude from the Table 4 that when the molar ratio between ASA and CS increased from 0.25 to 0.5, the EE and LC significantly enhanced. With the molar ratio between ASA and CS increasing from 0.5 to 0.75, the EE and LC slightly enhanced but had slight differences. However, when it increased from 0.75 to 1.5, the EE and LC of the ASA-CS nanoparticles had no significant changes, so it was advisable to choose 0.75 as molar ratio between ASA and CS.

### 3.6 Stability of ASA-CS nanoparticles

The morphological features and diameter, LC and in vitro release behavior of ASA-CS nanoparticles for long-term stability was observed by SEM, UV spectrophotometer. The results showed that morphological features and diameter, LC of ASA-CS nanoparticles had no obvious change and in vitro release behavior of them almost remained the same under the condition of 4 ± 1℃/60% RH± 5% RH, 25 ± 2℃/65% RH±5% RH and 40 ± 2^o^C/75% RH± 5% RH for 6 or 3 months, as shown in Table 5. All the data above mentioned demonstrated that ASA-CS nanoparticles had been proved to have high chemical stability against environmental changes.

**Table 5.** Stability studies- in vitro release of ASA-CS nanoparticles stored at 25 ± 2 ℃/60% RH± 5% RH, 30 ± 2 ℃/65% RH± 5% RH and 40 ± 2 ℃/75% RH± 5% RH in pH 7.4(after 0, 2, 4 & 6 weeks storage).

Time (h)	Cumulative drug release(%)											
	5 ± 2℃/60% RH± 5% RH				25 ± 2℃/65% RH± 5% RH				40 ± 2℃/75% RH± 5% RH			
	0	3	6	9	0	3	6	9	0	1	2	3
0	0	0	0	0	0	0	0	0	0	0	0	0
1	36.14	35.46	36.24	35.91	36.14	35.66	36.19	36.06	36.14	35.46	36.24	35.89
2	45.21	45.39	44.98	45.03	45.19	45.71	45.03	45.03	45.21	45.42	45.01	44.97
4	55.19	55.36	54.72	55.22	55.21	55.36	54.73	55.31	55.19	55.33	54.26	55.37
6	67.65	67.11	66.81	67.24	67.76	67.15	66.81	67.42	67.65	67.14	66.72	67.24
8	76.96	77.01	76.83	77.21	76.96	77.11	76.83	77.31	76.96	69.96	76.79	77.27
10	80.63	79.66	79.38	79.82	80.66	79.66	79.42	79.83	80.63	80.03	79.38	79.51
12	82.31	82.55	81.86	82.49	82.31	82.55	81.91	82.49	82.31	82.61	81.82	82.52
24	86.22	86.91	86.43	86.19	86.32	86.79	86.43	86.29	86.22	86.83	86.61	86.31
36	85.97	85.81	86.03	86.12	86.02	85.91	86.02	86.12	85.97	85.83	86.03	86.22

### 3.7 In vitro release studies of ASA-CS nanoparticles

**Figure 6.**
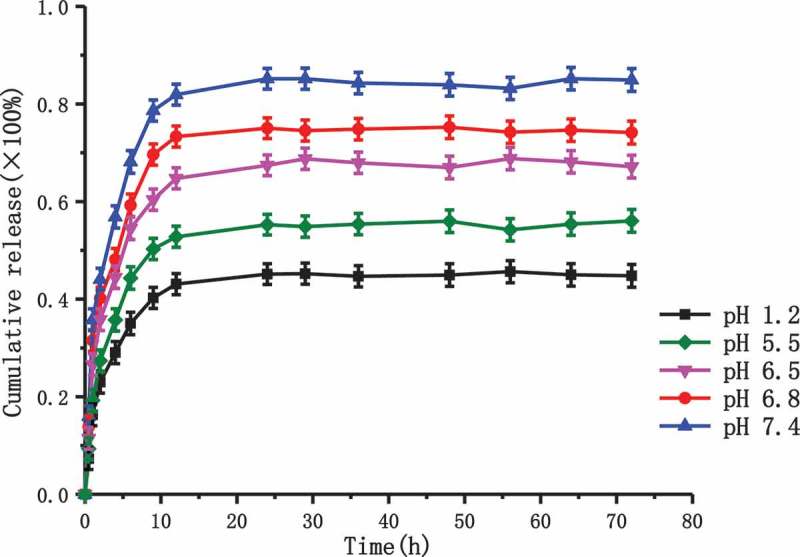
The release curve of ASA/CS nanoparticles in *vitro.*

The in *vitro* cumulative release profiles of ASA from ASA-CS nanoparticles in different pH value of release medium were exhibited in Figure 6. We could find from Figure 6 that the release profiles appeared to have three phases. The first phase was characterized by a rapid release or burst release which was mainly due to the fact that the ASA molecules on the surface of nanoparticles were swiftly diffused as a result of strong interaction of ASA with the media within the first 4 h. The second phase was characterized by a slower release process than in the first phase for times ranging from 4 to 12 h, which could be caused by the ASA was slowly released by the water-soluble pore of the carrier material in the diffusion way for 12 h after the dissolution of ASA in release medium to enter the nanoparticle caused by the dissolution of polymer. The third phase was characterized by a slowest release process among the three phases due to the polymer degradation. The dissolved drugs diffused into the release medium were observed. The results showed that the cumulative percentage release of the ASA from the nanoparticles was about 83% in the medium of pH 7.4, while 44% ASA were in the medium of pH 1.2 and 55%, 67%, 74% ASA were in the medium of pH 5.5, 6.5, 6.8 respectively. All of the results above indicated that the cumulative percentage release of the ASA was significantly and positively correlated with the pH value of the medium and ASA was easily released in slightly alkaline condition.

### 3.8 Effect of ASA-CS nanoparticles on carotid artery thrombosis in rats

**Figure 7.**
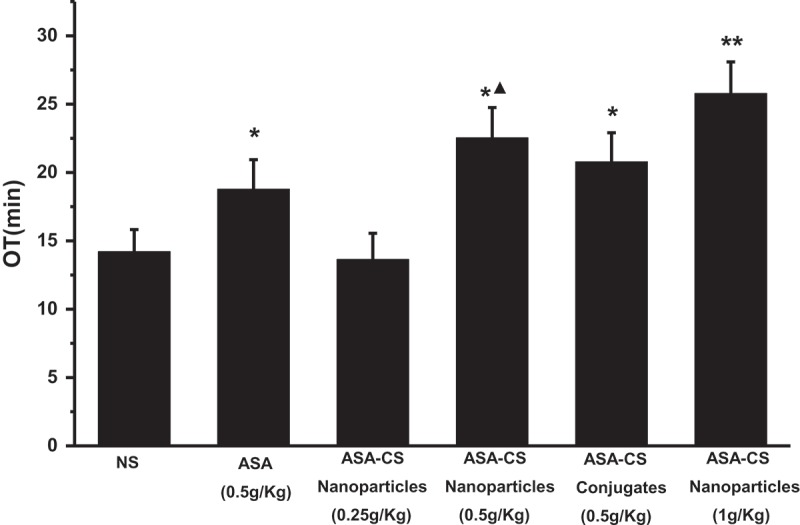
Effect of *po* ASA/CS nanoparticles (0.25, 0.5, 1.0g/Kg) on carotid artery thrombosis in rats. All values are expressed as means ± SD, *P < 0.05, **P < 0.01 vs control, ^▲^*P* < 0.05 vs ASA(0.5g/Kg) (n = 10).

Figure 7 showed that *po* 0.25 g/Kg ASA-CS nanoparticles had no significant effects (*P ***< 0.05**) on prolonging the occlusion time of carotid artery thrombosis compared with the control group, but *po* 0.5 g/Kg ASA-CS nanoparticles and ASA-CS conjugates had significant effects, and the influence increased with the rise of dose. The results above suggested that ASA-CS nanoparticles could affect the carotid artery thrombosis in dose-dependent relationship. At the time, *po* 0.5 g/Kg ASA had significant effects (*P ***< 0.05**) on prolonging the occlusion time of carotid artery thrombosis compared with the control group，and 0.5 g/Kg ASA-CS nanoparticles had more obvious influence on prolonging OT than that of ASA at corresponding dose (0.5 g/Kg) and 0.5g/Kg ASA-CS conjugates also, but less than ASA-CS nanoparticles at corresponding dose (1 g/Kg). From what had been discussed above, it could conclude that the own production ASA-CS nanoparticles would make a very good clinical effect for the treatment of thrombosis.

## 4. Conclusions

In this study, ASA/CS copolymer was synthesized by interpolymer complexation methods with mean diameter of 79.3 ± 24.6 nm. The characterization experiments showed that the carboxyl group of the ASA had firmly integrated on the amino group of CS and the ASA/CS surface was smooth, dense and near-spherical. The prepared ASA/CS nanoparticles showed high stability against environmental changes. The results from different experimental conditions on the properties of ASA-CS nanoparticles indicated that ASA-CS nanoparticles had satisfactory LC and EE as 27.27% and 46.88%，respectively. In vitro releasing studies showed that ASA could be easily released in slightly alkaline condition. Preliminary pharmacology experiment exhibited that the significant prolonging of OT of ASA-CS nanoparticles than that of ASA and ASA-CS conjugates at corresponding dose. All the results indicated that ASA/CS nanoparticles may have promising pharmaceutical application, and further pharmacological research is needed to confirm these beneficial effects.
